# Male sex and history of ischemic heart disease are major risk factors for anastomotic leakage after laparoscopic anterior resection in patients with rectal cancer

**DOI:** 10.1186/s12876-018-0846-3

**Published:** 2018-07-17

**Authors:** Seiichi Shinji, Yoshibumi Ueda, Takeshi Yamada, Michihiro Koizumi, Yasuyuki Yokoyama, Goro Takahashi, Masahiro Hotta, Takuma Iwai, Keisuke Hara, Kohki Takeda, Mikihiro Okusa, Hayato Kan, Eiji Uchida, Hiroshi Yoshida

**Affiliations:** 1 Department of Gastrointestinal and Hepato-Biliary-Pancreatic Surgery, Nippon Medical School, 1-1-5 Sendagi, Bunkyo-ku, Tokyo, 113-8603 Japan; 2 Graduate School of Arts and Sciences, The University of Tokyo, 3-8-1 Komaba, Meguro-ku, Tokyo, 153-8902 Japan; 3 AMED-PRIME, Japan Agency for Medical Research and Development, Tokyo, Japan

**Keywords:** Surgical complication, Preoperative creatinine, Double stapling technique, Diverting ileostomy

## Abstract

**Background:**

Anastomotic leakage (AL) is the most serious and common complication of surgery for rectal cancer, and associated risk factors remain unknown despite developments in laparoscopic surgery. The present study aimed to determine risk factors for AL after laparoscopic anterior resection (AR) of rectal cancer.

**Methods:**

This retrospective cohort study extracted information from a prospective database of all consecutive colorectal resections that proceeded at Nippon Medical School Hospital between January 2011 and December 2015 (*n* = 865). We identified 154 patients with rectal cancer treated by elective laparoscopic AR with anastomosis using primary double-stapling. Clinical variables and comorbidity, habits, and surgery-related variables were assessed by univariate and multivariate analyses to determine preoperative risk factors for clinical AL.

**Results:**

The overall rate of clinical AL was 11.7% (18 of 154 patients), and 5 (27.8%) of 18 patients required revised laparotomy. Data from males were analyzed because AL occurred only in males. Univariate analysis of male patients (*n* = 100) significantly associated preoperative creatinine values (*p* = 0.03) and a history of ischemic heart disease (IHD) (*p* = 0.012) with AL. The frequency of AL tended to increase (*p* = 0.06) when patients had low AR (*p* = 0.06) and transanal drainage. Having AL significantly prolonged hospital stays compared with patients without leakage (36.2 vs. 11.1 days; *p* <  0.01). Multivariate analysis identified a history of IHD (odds ratio [OR], 4.73; 95% confidence interval [CI], 1.27–17.5; *p* = 0.025] as an independent risk factor for AL.

**Conclusions:**

Male sex and a history of IHD are possible risk factors for AL after elective laparoscopic rectal cancer surgery.

## Background

Anastomotic leakage (AL) is a complication that occurs in 1.2 to 19.0% of patients during anterior resection (AR) for rectal cancer [1–11]. This complication can lead to serious conditions such as peritonitis and sepsis, repeated surgeries or percutaneous intervention with prolonged hospitalization, increased cost [4, 6–9], and a worse oncological prognosis [12, 13]. The basic requirements for anastomotic healing are an appropriate blood supply, healthy bowel ends, and tension-free anastomosis [14]. Risk factors for AL after AR have been discussed since anastomosis was initially established. Reported risk factors during open surgery include surgical duration, amount of intraoperative hemorrhage, and amount of blood transfusion [15, 16].

The number of elderly patients with colon cancer has increased along with the aging of society. Elderly patients often have co-morbidities and age-related physiological problems that can lead to worse postoperative complications compared with younger patients [17]. Risk factors for colon surgery should be re-evaluated depending on changes in social situation.

Laparoscopic surgery for rectal cancer can eliminate blind areas in the narrow surgical field of the pelvis, and rectal surgery can now proceed in a magnified operative field, thus improving the quality of this procedure, although laparotomy has historically contributed to the treatment of diseases of the digestive system because it allows complete visualization of the pelvis [18, 19]. Despite recent progress in laparoscopic surgery and standardized surgical technique [1–3, 20–27], some patients still develop AL. Little is known about risk factors for AL after laparoscopic rectal surgery. The present study aimed to determine risk factors for AL based on patients’ characteristics, extent of tumor progression, and factors related to surgery.

## Methods

### Study design and variables

**Fig. 1:**
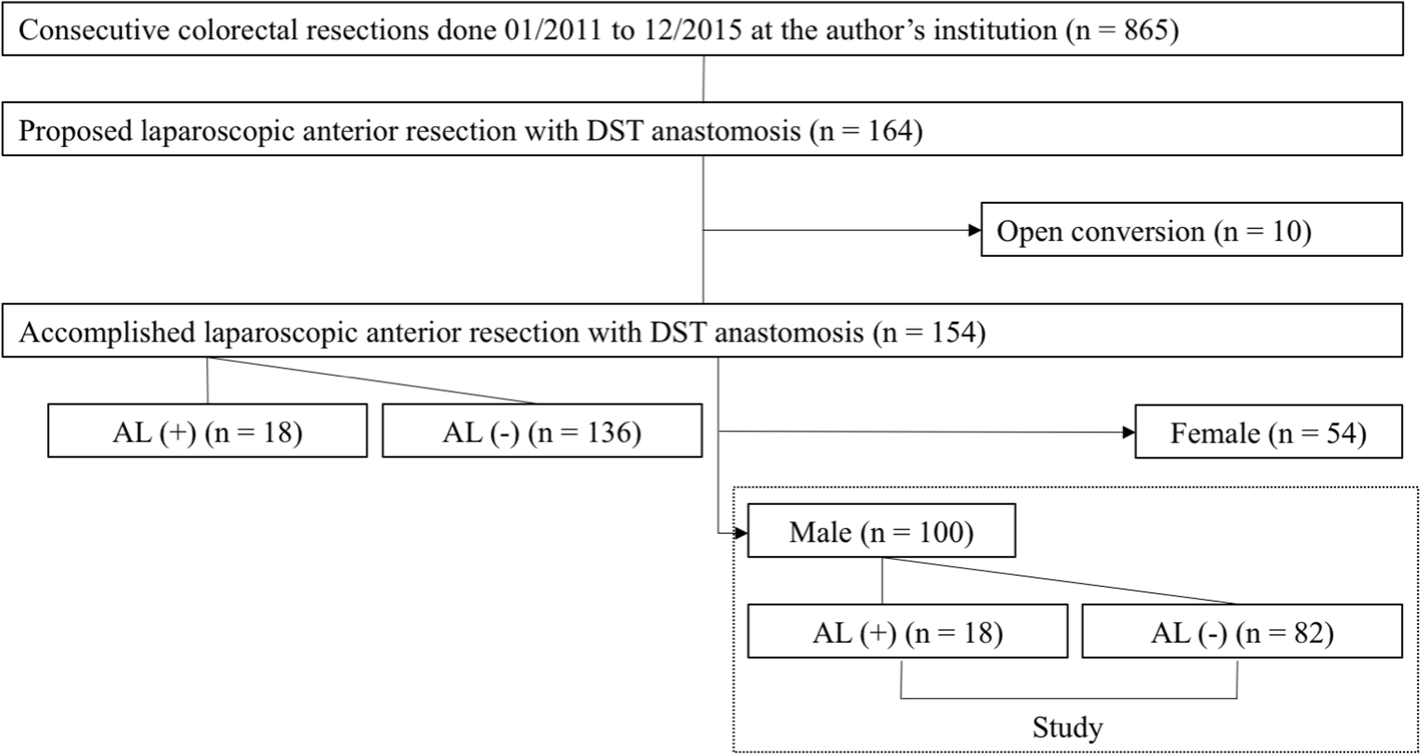
Flow chart and methodology

Figure 1 shows a flow chart of the methodology. We performed colorectal cancer surgery on 865 patients at Nippon Medical School Hospital between January 2011 and December 2015. Among these, 164 consecutive patients underwent elective laparoscopic high anterior resection (HAR) or low anterior resection (LAR) with anastomosis using double stapling (DS). After excluding 10 patients who had been converted to laparotomy, 154 patients were included in this study. Anastomotic leakage diagnosed at the discretion of the providing surgeon by clinical and/or radiological means, was classified into five grades using the Clavien-Dindo (CD) system [28]. We included symptomatic AL, which required active therapeutic intervention or reoperation (CD ≥ 2) for primary endpoint analysis. Tumor location was classified as being in the upper (distal border of tumor 10–15 cm from the anal verge), middle (5–10 cm), and lower rectum (≤ 5 cm) [1], based on perioperative confirmation of the preoperative findings of barium enemas, colonoscopy and pelvic computed tomography (Tables 1 and 2).

**Table 1:** Characteristics of the Patients

Parameter	Total	Anastomotic leakage (+)	Anastomotic leakage (−)	*p*
Number of patients	154	18	136	
Gender, m/f, n (%)	100/54 (65/35)	18/0 (100/0)	82/54 (60/40)	0.0003
Age, yr., mean ± SD (median, range)	67.1 ± 11.0 (69, 36–87)	64.7 ± 7.3 (63, 52–72)	67.4 ± 11.4 (70, 36–87)	0.12
BMI, kg/m^2^, mean ± SD (median, range)	23.0 ± 3.3 (22.9, 15.2–36.9)	23.5 ± 3.7 (22.9, 15.2–28.6)	22.9 ± 3.2 (22.9, 16.8–36.9)	0.27
BMI, ≥ 25, n (%)	38 (25)	8 (44)	30 (22)	0.08
ASA				1.00
1	22 (14)	2 (11)	20 (15)	
2	121 (79)	15 (83)	106 (78)	
3	11 (7)	1 (6)	10 (7)	
Tumor location (from the anal verge), n (%)				0.32
Upper (10–15 cm)	66 (43)	5 (28)	61 (45)	
Middle (5–10 cm)	56 (36)	9 (50)	47 (35)	
Lower (< 5 cm)	32 (21)	4 (13)	28 (21)	
Tumor size, mm, mean ± SD (median, range)	39.7 ± 20.4 (38, 7–170)	45.6 ± 19.3 (50, 7–80)	38.9 ± 20.4 (35, 8–170)	0.08
UICC-TNM Stage, n (%)				0.62
0	2 (1)	0 (0)	2 (1)	
I	46 (30)	6 (33)	40 (29)	
II	40 (26)	5 (28)	35 (26)	
III	45 (30)	3 (17)	42 (31)	
IV	20 (13)	4 (22)	16 (12)	
Unknown (CR case after preoperative chemotherapy)	1 (1)	0 (0)	1 (1)	
T category, n (%)				0.65
Tis	2 (1)	0 (0)	2 (1)	
T1	21 (14)	2 (11)	19 (14)	
T2	36 (23)	4 (22)	32 (24)	
T3	76 (49)	11 (61)	65 (48)	
T4	16 (10)	0 (0)	16 (12)	
N category, n (%)				0.96
N0	95 (62)	11 (61)	84 (62)	
N1	40 (26)	5 (28)	35 (26)	
N2	14 (9)	2 (11)	12 (9)	
N3	5 (3)	0 (0)	5 (4)	
M category, n (%)				0.26
M0	134 (87)	14 (78)	120 (88)	
M1	20 (13)	4 (22)	16 (12)	
Preoperative chemotherapy, n (%)	11 (7)	1 (6)	10 (7)	1.00
Preoperative decompression, n (%)	2 (1)	0 (0)	2 (1)	1.00
WBC, × 10^2^/μl, mean ± SD	63.5 ± 16.3	69.7 ± 15.2	62.7 ± 16.3	0.09
Plt, × 10^4^/μl, mean ± SD	24.5 ± 6.7	26.4 ± 10.6	24.2 ± 6.0	0.19
Creatinine, mg/dl, mean ± SD	0.83 ± 0.28	1.05 ± 0.51	0.80 ± 0.23	< 0.001
Total cholesterol, mg/dl, mean ± SD	192 ± 38	177 ± 52	194 ± 36	0.10
Triglyceride, mg/dl, mean ± SD	122 ± 65	128 ± 68	122 ± 65	0.72
Total protein, g/dl, mean ± SD	7.5 ± 5.4	7.0 ± 0.4	7.5 ± 5.8	0.70
Albumin, g/dl, mean ± SD	4.4 ± 3.6	3.9 ± 0.5	4.4 ± 3.8	0.58
Blood sugar, mg/dl, mean ± SD	111 ± 33	127 ± 44	109 ± 31	0.06
Any comorbidity, n (%)	114 (74)	16 (89)	98 (72)	0.16
Hypertension, n (%)	74 (48)	9 (50)	65 (48)	1.00
Ischemic Heart Disease, n (%)	13 (8)	6 (33)	7 (5)	0.001
Arrhythmia, n (%)	6 (4)	1 (6)	5 (4)	0.47
Cerebrovascular Disease, n (%)	9 (6)	3 (17)	6 (4)	0.07
Asthma, n (%)	5 (3)	1 (6)	4 (3)	0.47
COPD, n (%)	5 (3)	0 (0)	5 (4)	1.00
Diabetes Mellitus, n (%)	37 (24)	7 (39)	30 (22)	0.14
Dyslipidemia, n (%)	28 (18)	1 (6)	27 (20)	0.20
Smoking, n (%)	33 (21)	4 (22)	29 (21)	1.00
The use of antiplatelet and/or anticoagulant agent, n (%)	28 (18)	6 (33)	22 (16)	0.10
Type of operation				0.02
High anterior resection	44 (29)	1 (6)	43 (32)	
Low anterior resection	110 (71)	17 (94)	93 (68)	
High tie, n (%)	98 (64)	9 (50)	89 (65)	0.21
D				0.42
2	47 (30)	7 (39)	40 (29)	
3	107 (70)	11 (61)	96 (71)	
LN harvested, mean ± SD (median, range)	15.8 ± 7.6 (15, 2–40)	13.8 ± 5.3 (13, 5–23)	16.0 ± 7.9 (16, 2–40)	0.42
LN metastasized, mean ± SD (median, range)	1.6 ± 3.5 (0, 0–23)	1.4 ± 2.7 (0, 0–11)	1.6 ± 3.6 (0, 0–23)	0.97
Cur				0.20
A	134 (87)	14 (78)	120 (88)	
B	4 (3)	0 (0)	4 (3)	
C	16 (10)	4 (22)	12 (9)	
Number of stapler firings, mean ± SD (median, range)	1.3 ± 0.5 (1, 1–3)	1.5 ± 0.5 (1, 1–2)	1.3 ± 0.5 (1, 1–3)	0.06
Number of stapler firings, ≥ 2 times (%)	46 (30)	9 (50)	37 (27)	0.06
Air leak test, n (%)	124 (81)	15 (83)	109 (80)	1.00
Air leak, air leak / air leak test (%)	4/124 (3)	1/15 (7)	3/109 (2)	0.33
Operative time, min, mean ± SD (median, range)	280 ± 93 (265, 134–692)	332 ± 118 (312, 160–631)	273 ± 87 (262, 134–692)	0.03
Post meridiem operation, n (%)	26 (17)	5 (28)	21 (15)	0.19
Operative blood loss, ml, mean ± SD (median, range)	84 ± 145 (25, 0–995)	131 ± 169 (60, 0–560)	78 ± 140 (20, 0–995)	0.07
Blood transfusion, n (%)	9 (6)	1 (6)	8 (6)	1.00
Temporary loop ileostomy, n (%)	23 (15)	2 (11)	21 (15)	1.00
Placement of transanal drain, n (%)	56 (36)	11 (61)	45 (33)	0.03
Postoperative stay, day, mean ± SD (median, range)	14.2 ± 11.6 (10, 7–77)	36.2 ± 19.4 (29, 10–77)	11.3 ± 5.7 (10, 7–43)	< 0.01

**Table 2:** Clinical and Pathological Characteristics of the Patients (Male)

Parameter	Anastomotic leakage (+)	Anastomotic leakage (−)	*p*
	*n* = 18	*n* = 82	
Age, yr., mean ± SD (median, range)	64.7 ± 7.3 (63, 52–79)	66.1 ± 11.2 (68, 38–87)	0.38
BMI, kg/m^2^, mean ± SD (median, range)	23.5 ± 3.7 (22.9, 15.2–28.6)	23.3 ± 3.2 (23.1, 16.8–33.6)	0.58
BMI, ≥ 25, n (%)	8 (44)	21 (26)	0.15
ASA			1.00
1	2 (11)	11 (13)	
2	15 (83)	64 (78)	
3	1 (6)	7 (9)	
Tumor location (from the anal verge), n (%)			0.49
Upper (10–15 cm)	5 (28)	35 (43)	
Middle (5–10 cm)	9 (50)	32 (39)	
Lower (< 5 cm)	4 (13)	15 (18)	
Tumor size, mm, mean ± SD (median, range)	45.6 ± 19.3 (50, 7–80)	39.7 ± 22.3 (35, 8–170)	0.14
UICC-TNM Stage, n (%)			0.71
0	0 (0)	1 (1)	
I	6 (33)	23 (28)	
II	5 (28)	25 (30)	
III	3 (17)	22 (27)	
IV	4 (22)	11 (13)	
T category, n (%)			0.56
Tis	0 (0)	1 (1)	
T1	2 (11)	11 (13)	
T2	4 (22)	20 (24)	
T3	11 (61)	40 (49)	
T4	0 (0)	10 (12)	
N category, n (%)			0.87
N0	11 (61)	53 (65)	
N1	5 (28)	20 (24)	
N2	2 (11)	6 (7)	
N3	0 (0)	3 (4)	
M category, n (%)			0.46
M0	14 (78)	71 (87)	
M1	4 (22)	11 (13)	
Preoperative chemotherapy, n (%)	1 (6)	7 (9)	1.00
Preoperative decompression, n (%)	0 (0)	2 (2)	1.00
WBC, ×10^2^/μl, mean ± SD	69.7 ± 15.2	64.6 ± 15.4	0.21
Plt, ×10^4^/μl, mean ± SD	26.4 ± 10.6	23.6 ± 5.7	0.13
Creatinine, mg/dl, mean ± SD	1.05 ± 0.51	0.89 ± 0.21	0.03
Total cholesterol, mg/dl, mean ± SD	177 ± 52	187 ± 30	0.31
Triglyceride, mg/dl, mean ± SD	128 ± 68	124 ± 62	0.81
Total protein, g/dl, mean ± SD	7.0 ± 0.4	7.8 ± 7.5	0.65
Albumin, g/dl, mean ± SD	3.9 ± 0.5	4.6 ± 4.9	0.54
Blood sugar, mg/dl, mean ± SD	127 ± 44	113 ± 35	0.20

All patients scheduled for elective procedures underwent preoperative bowel preparation with oral laxatives two days before surgery and a glycerin enema without polyethylene glycol electrolyte solution on the day of the procedure. A pelvic drain was routinely placed behind the anastomosis and a transanal drain was placed according to the status of the patient and the judgment of each surgeon. Patients started to intake fluids orally on the day after surgery and consume oral foods from postoperative day 3. Transanal drains were removed at 4–6 days after surgery after confirming the absence of signs of AL.

Study: Several factors were compared between patients with (*n* = 18) and without (*n* = 136) AL. Because all patients with AL were male, we compared the same factors between male patients with (n = 18) and without (*n* = 82) AL.

### Analyzed factors

The following variables were included in analyses: sex, age, body mass index (BMI), American Society of Anesthesiologists (ASA), tumor location, tumor size, UICC-TNM stage (7th edition), preoperative chemotherapy, preoperative decompression, laboratory findings, comorbidities, tobacco use, antiplatelet and/or anticoagulant agents, type of surgical procedure (HAR or LAR at the upper or lower side of peritoneal reflection, respectively), level of inferior mesenteric artery (IMA) ligation (high or low tie), D number (extent of lymph node dissection; D0, incomplete dissection of perirectal lymph nodes; D1, complete dissection of perirectal lymph nodes; D2, complete dissection of perirectal and intermediate lymph nodes; D3, complete dissection of all regional lymph nodes), number of lymph nodes harvested, number of lymph node metastases, curability (Cur: A, R0 in stages 0, I, II or III, B, R0 in stage IV or R1 in any stage; C, R2 in any stage), number of stapler firings, positive air leak test, surgical duration, afternoon surgical procedure, intraoperative blood loss, intraoperative blood transfusion, temporary loop ileostomy, transanal drainage, and length of postoperative stay. Our patients were not subjected to radiation treatments.

### Statistical analyses

All data were statistically analyzed using EZR (Saitama Medical Center, Jichi Medical University, Saitama, Japan), a graphical user interface for R (The R Foundation for Statistical Computing, Vienna, Austria) that is a modification of R commander designed to add functions frequently applied in biostatistics [29]. Categorical variables were compared and analyzed using chi-square tests, Fisher’s exact tests and Mann-Whitney U tests. Quantitative data are presented as means ± standard deviation (SD) and compared using Mann-Whitney U tests. All analyses were two-sided, and a *p* value of < 0.05 was considered statistically significant. Factors associated with AL were determined using multivariate logistic regression analysis and factors with *p* <  0.10 and age were included in the model.

## Results

### Patient population

Table 1 shows the clinical characteristics, comorbidities and habits, and surgery-related factors among all 154 patients (male, *n* = 100 [65%]; median age, 69 years; range, 36–87 years) who were treated for rectal cancer between January 1, 2011 and December 31, 2015. Their median BMI was 22.9 (range, 15.2–36.9) kg/m^2^ and 25% were obese (BMI ≥ 25 kg/m^2^). A total of 66 (43%), 56 (36%), and 32 (21%) patients had cancer of the upper, middle, and lower rectum, respectively. Anastomotic leakage generally occurs more frequently at the lower, than the upper rectum (Table 1). However, this study did not find any statistically significant differences in leakage among locations. The average size of tumors was 39.7 (median, 38; range, 7–170) mm. The UICC-TNM stage of one patient who achieved a complete response after preoperative chemotherapy was classified as unknown. Eleven (7%) patients underwent preoperative chemotherapy comprising six courses of modified FOLFOX6. Two (1%) patients required decompression due to obstruction.

A total of 114 (74%) patients had comorbidities; 74 (48%) had hypertension, 13 (8%) had a history of ischemic heart disease (IHD; angina pectoris, *n* = 7; acute myocardial infarction, *n* = 6), 6 (4%) had arrhythmia, 9 (6%) had a history of cerebrovascular disease, 5 (3%) had asthma, 5 (3%) had chronic obstructive pulmonary disease (COPD), 37 (24%) had diabetes mellitus, and 28 (18%) had dyslipidemia. Fifteen patients smoked and 28 (18%) used antiplatelet and/or anticoagulant agents.

Among the patients, 29 and 71% underwent HAR and LAR, respectively, 98 (64%) underwent ligation at the root of the IMA (high ligation) and 107 (70%) underwent complete dissection of all regional lymph nodes (D3 dissection). A mean of 15.8 (median, 15; range, 2–40) lymph nodes was harvested and an average of 1.6 (median, 0; range, 0–23) were metastatic. The surgery was curative in 134 (87%) patients and 108 (70%) and 46 (30%) underwent rectal transection using one, and two or more stapler cartridges, respectively. Air leaks detected in 4 (3%) of 124 (81%) patients were addressed using intracorporeal reinforcing sutures. The average surgical duration was 280 (median, 265; range, 134–692) min and 26 (17%) underwent procedures. The average operative blood loss was 84 (median, 25; range, 0–995) mL. Nine (6%) patients required blood transfusions during surgery. A temporary loop ileostomy was constructed in 23 (15%) patients, and transanal drains were placed in 56 (36%). The mean postoperative hospital stay was 14.2 (median, 10; range, 7–77) days.

Among these patients, 18 (11.7%) had AL (Table 1) with a CD classification of > 2. Anastomotic leaks were found only in male patients, among whom 13 (72.2%) did not require a repeat procedure (CD classification 2 or 3a) and 5 (27.8%) did (CD classification 3b and 4). Among 18 patients, 1, 1, 4, 4, 2, 1, 2, 1, 1, and 1 developed anastomotic leaks on postoperative days 1, 2, 3, 4, 5, 6, 7, 10, 11 and 17, respectively. All leaks were clinically judged based on evidence of the extravasation of bowel contents through the drains, and the extent of intra-abdominal collection adjacent to the anastomosis was evaluated by computed tomography. The surgeons decided the timing of drain removal. Four patients were judged positive on intraoperative air leak tests, and all received intracorporeal reinforcing sutures. One of the four patients underwent ileostomy, and CD classification 2 AL occurred in another patient.

**Fig. 2:**
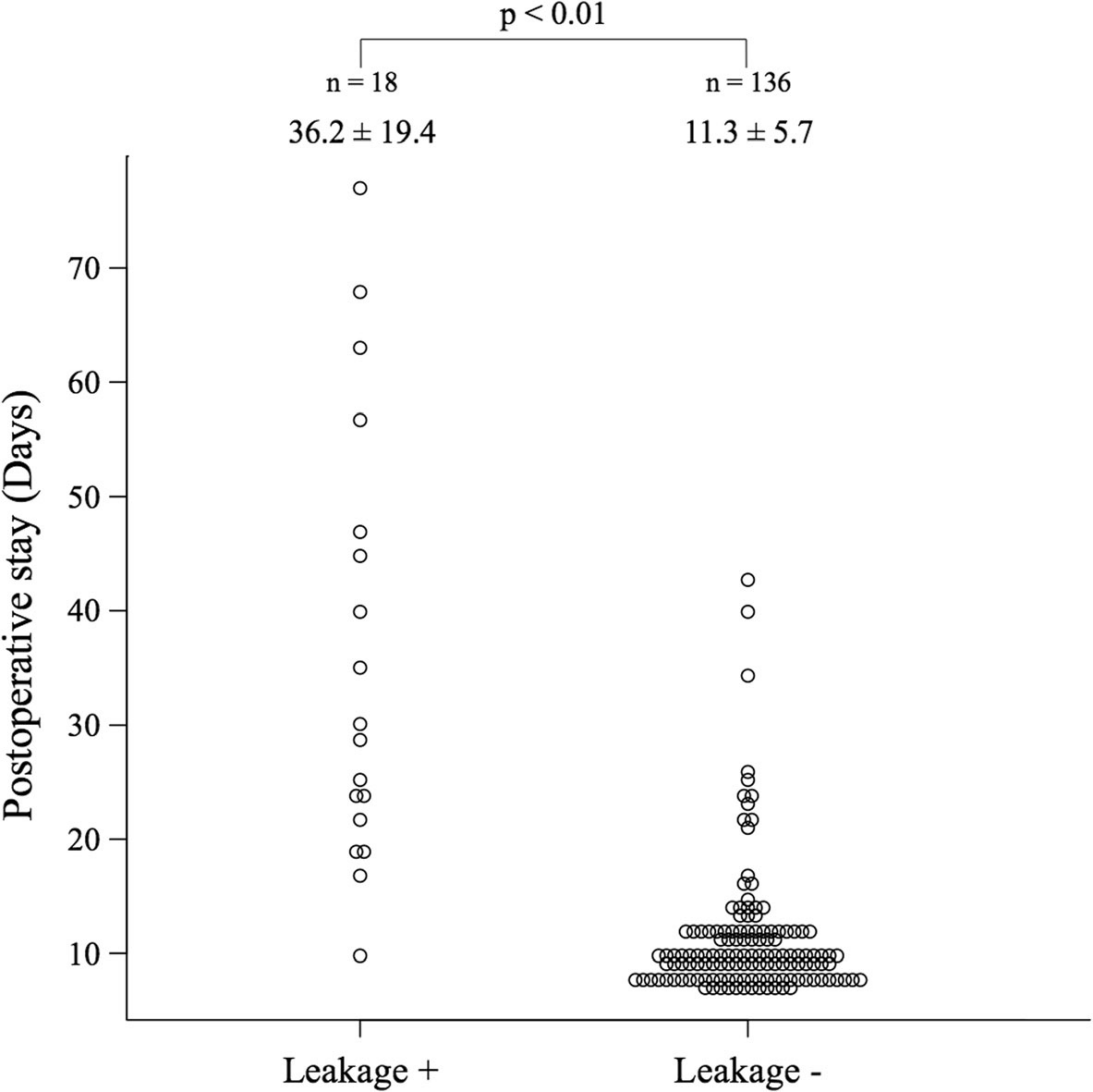
Comparison of hospital stays between patients with and without anastomotic leakage. Anastomotic leakage (AL) significantly prolonged hospital stays

We classified the patients based on whether they had anastomotic leakage CD ≥ 2 (Fig. 1). Clinical variables, comorbidities, habits, and surgery-related factors are summarized in Table 1. Univariate analysis selected male sex (*p* = 0.0003), preoperative creatinine value (*p* <  0.001), history of IHD (*p* = 0.001), LAR (*p* = 0.02), longer surgical duration (*p* = 0.03), and transanal drainage (*p* = 0.03) as significant risk factors. Notably, BMI ≥ 25 kg/m^2^ (*p* = 0.08), tumor size (*p* = 0.08), white blood cell (WBC) (*p* = 0.09), blood sugar (*p* = 0.06), history of cerebrovascular disease (*p* = 0.07), > two firings for rectal transection (*p* = 0.06), and a large volume of operative blood loss (*p* = 0.07) tended to correlate with AL. The development of AL significantly prolonged hospital stays (Fig. 2).

Because CD ≥ 2 AL did not occur in females, we focused on the 18 (18%) of 100 male patients who developed CD ≥ 2 AL. Tables 2, 3, and 4 show the clinical variables, comorbidities and habits, and surgery related factors, respectively. Univariate analysis selected preoperative creatinine value (*p* = 0.034) and a history of IHD (*p* = 0.012) as significant risk factors. Transanal drainage and LAR tended to correlate with AL (both *p* = 0.06). Multivariate analyses that included the predictors of AL selected in the univariate analysis (*p* <  0.10) and the patients’ age, showed that IHD (odds ratio [OR], 4.73; 95% confidence interval [CI], 1.27–17.5; *p* = 0.025) remained a statistically significant independent predictor of AL after laparoscopic AR (Table 5).

**Table 3:** Comorbidity and habit (Male)

Parameter	Anastomotic leakage (+)	Anastomotic leakage (−)	*p*
	n = 18	n = 82	
Any comorbidity, n (%)	16 (89)	62 (76)	0.35
Hypertension, n (%)	9 (50)	38 (46)	0.80
Ischemic Heart Disease, n (%)	6 (33)	7 (9)	0.012
Arrhythmia, n (%)	1 (6)	4 (49)	1.00
Cerebrovascular Disease, n (%)	3 (17)	6 (7)	0.20
Asthma, n (%)	1 (6)	2 (2)	0.45
COPD, n (%)	0 (0)	3 (4)	1.00
Diabetes Mellitus, n (%)	7 (39)	23 (28)	0.40
Dyslipidemia, n (%)	1 (6)	14 (17)	0.30
Smoking, n (%)	4 (22)	14 (17)	0.78
The use of antiplatelet and/or anticoagulant agent, n (%)	6 (33)	20 (24)	0.55

**Table 4:** Surgery-related factor (Male)

Parameter	Anastomotic leakage (+)	Anastomotic leakage (−)	*p*
	n = 18	n = 82	
Type of operation			0.06
High anterior resection	1 (6)	22 (27)	
Low anterior resection	17 (94)	60 (73)	
High tie, n (%)	9 (50)	55 (67)	0.19
D			0.40
2	7 (39)	23 (28)	
3	11 (61)	59 (72)	
LN harvested, mean, mean ± SD (median, range)	13.8 ± 5.3 (13, 5–23)	15.9 ± 7.6 (15, 2–38)	0.44
LN metastasized, mean, mean ± SD (median, range)	1.4 ± 2.7 (0, 0–11)	1.3 ± 2.7 (0, 0–15)	0.92
Cur			0.28
A	14 (78)	71 (87)	
B	0 (0)	4 (5)	
C	4 (22)	7 (8)	
Number of stapler firings, mean ± SD (median, range)	1.5 ± 0.5 (1, 1–2)	1.3 ± 0.5 (1, 1–3)	0.19
Number of stapler firings, ≥ 2 times (%)	9 (50)	27 (32)	0.19
Air leak test, n (%)	15 (83)	69 (84)	1.00
Air leak, air leak / air leak test (%)	1/15 (7)	1/69 (1)	0.33
Operative time, min, mean ± SD (median, range)	332 ± 118 (312, 160–631)	294 ± 90 (267, 156–692)	0.20
Post meridiem operation, n (%)	5 (28)	10 (12)	0.14
Operative blood loss, ml, mean ± SD (median, range)	131 ± 169 (60, 0–560)	86 ± 143 (30, 0–995)	0.22
Blood transfusion, n (%)	1 (6)	3 (4)	0.55
Temporary loop ileostomy, n (%)	2 (11)	11 (13)	1.00
Placement of transanal drain, n (%)	11 (61)	29 (35)	0.06
Postoperative stay, day, mean ± SD (median, range)	36.2 ± 19.4 (29, 10–77)	11.1 ± 4.7 (10, 7–34)	< 0.01

**Table 5:** Multivariate logistic regression analysis evaluating possible risk factors associated with anastomotic leak (Male)

Parameter	Odds ratio	95%CI	*p*
Age, year	0.98	0.92–1.04	0.49
Ischemic Heart Disease, yes vs no	4.73	1.27–17.5	0.025
Surgical procedure, LAR vs HAR	3.14	0.35–28.0	0.24
Creatinine, mg/dl	5.06	0.75–34.0	0.10
Placement of trans anal drain, yes vs no	2.52	0.73–8.7	0.14

## Discussion

The present study aimed to identify risk factors for AL after laparoscopic AR for rectal cancer. Several risk factors are reportedly associated with AL after open AR, but few studies have examined the frequency of AL after laparoscopic AR. Univariate and multivariate analyses uncovered male sex and a history of IHD as independent predictive factors for AL after laparoscopic AR. The risk of AL is 4.7-fold higher in patients with, than without IHD.

How a history of IHD affects AL is uncertain. Kruschewski et al. found that IHD is a risk factor for anastomotic leakage after open AR [30]. Intraoperative laser-Doppler flowmetry has shown that reduced blood flow at the rectal stump is associated with an increased risk of AL [31]. A basic study by Fawcett et al. histologically demonstrated a relationship between AL and serosal microvascular disease at anastomotic sites, indicating that defective microcirculation reduces blood flow and leads to poor wound healing [32]. Considering that IHD is associated with arteriosclerosis, our findings suggest that patients with a history of IHD already have intestinal microvascular disease, which disrupts circulation at anastomotic sites. This notion remains to be proven from a pathological perspective. However, understanding the mechanism of AL development might contribute to the discovery or development of drugs to prevent AL.

Univariate analysis revealed significantly higher preoperative serum creatinine values in patients with AL. Although significance was not reached in the multivariate analysis (*p* = 0.01), confounders might have excluded preoperative creatinine value as a risk factor. However, we believe that the preoperative creatinine level is important with respect to postoperative AL in patients with rectal cancer. Kidney dysfunction is a common risk factor for IHD and arteriosclerosis [33, 34], and elevated serum creatinine is likely to indirectly indicate degrees of mesenteric microcirculatory dysfunction. Additionally, deteriorating drug metabolism/excretion and tissue edema from a water-electrolyte imbalance associated with kidney dysfunction might result in poor wound healing, which in turn can lead to AL [35]. Other risk factors comprised the LAR surgical procedure and transanal drainage (both *p* = 0.06). One published article describes a lower location of anastomosis as a risk factor for leakage [36]. The male pelvis is generally narrower towards the anus than that of females, which renders LAR more technically difficult in general. However, LAR was not an independent risk factor in the present study. We constructed diverting ileostomies more often after LAR than HAR (16.9% vs. 0%, *p* = 0.036; data not shown), because LAR is a substantial risk factor for AL in males. The only risk factor for anastomotic leakage in the present study was IHD. Among lifestyle diseases such as hyperlipidemia, diabetes, hypertension, and smoking that are closely associated with IHD, only smoking has been shown to be a risk factor for anastomotic leakage [30, 37]. However, the present analyses did not select smoking or any other lifestyle diseases as risk factors for anastomotic leakage. Thus, we reduced anastomotic leakage in patients with a history of IHD by creating a stoma.

Criteria for where a transanal drain is placed are not defined at our institution and transanal drainage is applied at the discretion of the surgeon. Whether or not to attach a transanal drain is controversial. Transanal drains are simple to attach and detach, which relieves patients of the need for prolonged surgery, but bowel contents cannot be removed from the intestine. In contrast, loop ileostomy prevents anastomotic leakages from worsening. However, complications that can arise due to loop ileostomy placement include outlet obstruction and stoma detachment. In addition, surgical stoma closure under general anesthesia confers additional stress on patients. A meta-analysis has shown that transanal tube positioning helps to lower the postoperative incidence of AL, hence reducing the necessity for subsequent reoperation [38]. In contrast, the AL rate was significantly higher in 11 (61%) of the 18 patients who required transanal drainage in the present study. These results suggest that AL cannot be prevented in a population of patients only by postoperative rectal decompression. Conversely, this finding might indicate that surgeons empirically identified patients who were likely to have AL at baseline, which in turn might have led to the more frequent application of transanal drainage in the AL group. However, to conclude from these results that transanal drainage is ineffective seems somewhat risky. This is because among the 45 patients who had transanal drainage but no AL, some of them might be able to avoid AL with transanal drainage without the need for a diverting ileostomy.

Finally, more elderly individuals tend to develop colorectal cancer, reflecting the recent aging of the Japanese population. The number of elderly patients with colorectal cancer accompanied by serious comorbidities including IHD is likely to further increase in the future. Therefore, selection of the surgical LAR method with a temporary stoma or the Hartmann procedure should be carefully considered.

## Conclusions

At a rate of 11.6%, AL remains a common and serious complication of curative surgery for rectal cancer. The present study determined that males with a history of IHD were at high risk of AL after AR. Thus, a temporary stoma or the Hartmann procedure should be considered. Uncovering the mechanism of AL in such patients might lead to the development of innovative drugs that could prevent AL and reduce the need to construct permanent stoma.

## Abbreviations

AL: Anastomotic leakage
AR: Anterior resection
ASA: American Society of Anesthesiologists
BMI: Body mass index
CD: Clavien-Dindo
CI: Confidence interval
COPD: Chronic obstructive pulmonary disease
Cur: Curability
DS: Double stapling
HAR: High anterior resection
IHD: Ischemic heart disease
IMA: Inferior mesenteric artery
LAR: Low anterior resection
OR: Odds ratio
PET: Positron emission tomography
SD: Standard deviation
